# Editorial: Between Theory and Clinic: The Contribution of Neuroimaging in the Field of Consciousness Disorders

**DOI:** 10.3389/fneur.2019.00165

**Published:** 2019-02-27

**Authors:** Olivia Gosseries, Caroline Schnakers, Steven Laureys

**Affiliations:** ^1^Coma Science Group, University Hospital of Liege, Liege, Belgium; ^2^GIGA-Consciousness, University of Liege, Liege, Belgium; ^3^Research Institute, Casa Colina Hospital and Centers for Healthcare, Pomona, CA, United States

**Keywords:** disorders of consciousness, unresponsive wakefulness syndrome/vegetative state, minimally conscious state, neuroimaging, electrophysiology

Patients surviving severe brain injury either recover quickly from coma or go through prolonged disorders of consciousness (DOC) such as unresponsive wakefulness syndrome (UWS) or minimally conscious state (MCS). While patients in MCS show signs of consciousness, patients in UWS only show reflexive behaviors. These patients are unable to communicate and present vigilance fluctuation, language impairments, and severe sensory-motor deficits. Once patients recover functional communication or use of objects, they are emerged from MCS (EMCS).

Even though behavioral assessment remains the gold standard for diagnosis, a number of studies highlight the difficulty in making the distinction between conscious and unconscious patients based on clinical examinations. Misdiagnosis can have serious consequences on patient's management, medically and ethically (i.e., regarding end-of-life decision).

The emergence of neuroimaging and neurophysiological techniques opened new opportunities to complement bedside assessment by improving diagnosis and prognosis of patients with DOC. This Research Topic, which includes 15 articles with 100 contributors, aims to give an overview on how neuroimaging research can improve diagnosis, prognosis and management of patients with DOC, and how recent applications of neuroimaging can help understand consciousness through severe brain injuries.

Vanhaudenhuyse et al. report the case of a misdiagnosis in a patient who was considered in an unresponsive wakefulness syndrome for 20 years. Repeated behavioral examinations (using the Coma Recovery Scale-Revised, CRS-R) combined with neuroimaging techniques (using positron emission tomography—PET, and magnetic resonance imaging—MRI) showed that the patient was in fact conscious and was re-diagnosed as an incomplete locked-in syndrome. Sarà et al. reported that some locked-in patients present hallucinations, and in a subgroup of 5 patients with such symptoms, they showed a reduced cortical volume in the right parahippocampal cortex, the fusiform and lingual regions, suggesting that advanced neuroimaging might help to detect small cortical changes.

In parallel, Aubinet et al. show how neuroimaging can help develop more sensitive behavioral tools to assess cognition in DOC patients. They describe the behavioral and cognitive profiles of 5 patients in MCS/EMCS alongside their neuroimaging results using structural MRI and PET. They introduce a new language-based neuropsychological tool, the *Cognitive Assessment by Visual Election* (CAVE), and showed that the cognitive profiles of the patients were consistent with the underlying brain impairments. More specifically, the presence of residual visual, motor, and language comprehension functions was, respectively, associated with a relative preservation of occipital, motor, and temporo-angular cortex metabolism.

Using functional MRI (fMRI) command following tasks, Bodien et al. compared the rate of covert consciousness detection by hand squeezing and tennis playing motor imagery paradigms in 10 patients with traumatic DOC and 10 healthy subjects. They found that the tennis paradigm performed better in healthy subjects, but in patients, the hand squeezing paradigm detected command following with greater accuracy. The hand squeezing paradigm may therefore be a better classifier of command following in DOC patients.

Several articles also aimed at exploring new ways to detect consciousness in DOC patients. To investigate the potential for internal and external awareness in DOC patients, Haugg et al. assessed the juxtaposed relationship between the default mode network (DMN) and the fronto-parietal (dorsal attention and executive control) networks' functional time-courses. Patients who demonstrated fMRI command following showed greater differentiation between the DMN and dorsal attention network in response to movie viewing, compared to the resting condition. This effect was similar to healthy subjects and was driven by the movie's narrative. Conversely, this pattern was not present in DOC patients who showed no fMRI-based evidence of covert awareness. Naturalistic paradigms could therefore be used to dissociate between groups of DOC patients with and without covert awareness.

Dell'Italia et al. present a different framework to estimate fMRI network properties based on Exponential Random Graph Models (ERGM), which overcomes current methodological limitations. Longitudinal data in one patient who sustained a severe traumatic brain injury show that throughout recovery from coma, brain graphs vary in their natural level of connectivity. Separable temporal ERGM can characterize network dynamics over time and show the specific pattern of formation and dissolution of connectivity after coma.

To differentiate DOC patients, Riganello et al. measured the complexity index of the heart rate variability using non-linear multi-scale entropies in 16 MCS and 14 UWS patients. Higher complexity index values were observed in MCS compared to UWS patients with high discriminative power using machine learning. The complexity index also correlated with brain connectivity in the central autonomic networks assessed with resting state fMRI. These findings suggest that this “heart” index can provide an indirect way to monitor brain connectivity in DOC patients.

Naro et al. used functional transcranial doppler to differentiate between patients in MCS and UWS by assessing cerebral blood flow velocity (CBFV) during passive movement tasks. They observed group difference changes in CBFV with the pulsatility index in 21 patients with DOC and 25 healthy controls. This rapid and easy tool may allow to identify residual top-down modulation processes from higher-order cortical areas to sensory-motor integration networks related to the peripersonal space. Another potential technique that is at its infancy in the field of DOC is the functional near-infrared spectroscopy, a non-invasive optical and portable device. Rupawala et al. review its potential application to improve the accuracy of diagnoses and to provide new ways of communication.

A few authors tackled to better understand brain processing in DOC patients and therefore, in turn, improve diagnostic and prognostic techniques in such population. Using an hybrid PET/MR imaging, Cavaliere et al. investigated test-retest brain connectivity variability in three DOC patients. Using graph-theory and independent component analyses, they found differences between test-retest acquisitions affecting each network and each patient in a different way. Higher functional/metabolic correlation was measured in the MCS and EMCS patients compared to the UWS patient. Performing multiple acquisitions within the same session allow to assess temporal patterns of resting-state networks and improve characterization of DOC patients.

Longitudinal assessments using high-density electroencephalography (EEG) and CRS-R have also been performed on a longer time scale (with 3-monthly intervals) by Bareham et al., who showed that measures of EEG networks correlated with behavioral variations. EEG connectivity captured both stability and recovery of behavioral trajectories within and between patients. This highlights the feasibility of bedside EEG assessments in rehabilitation setting, which can complement clinical evaluation.

Bai et al. reviewed resting state EEG studies in DOC for diagnosis, prognosis, and brain interventions. Spectrum power, coherence, and entropy were the most frequently used features while power spectrum and functional connectivity had the best performance differentiating UWS from MCS and healthy subjects. Permutation entropy in the theta frequency also had high classification accuracy for differential diagnosis. Regarding prognosis, in their systematic review and meta-analysis of brain data and outcome in DOC, Kotchoubey and Pavlov report that oscillatory EEG responses, sleep spindles, and the somatosensory cortical response N20 were the best outcome predictors for DOC, whereas the poorest prognostic effects were fMRI responses to stimulation and P300. They however conclude that no practical recommendations on prognosis indicators can be given at this stage and they suggest several considerations to improve future (prognosis) studies: each group of patients should include at least 20 patients, perform blind assessments, use a flow chart to illustrate the procedure of patient selection, include the full list of measured variables, report the time since injury and the time of outcome, and describe all positive and negative results.

The last two articles refer to therapeutic options. Gottshall et al. evaluated a patient in chronic MCS who received central thalamic deep brain stimulation (CT-DBS). After 1 year of treatment discontinuation, reduced responsiveness was observed along with the abolishment of sleep spindles, marked downregulation of slow wave sleep delta power, and the return of alpha-delta sleep. The authors discuss the mechanism of sleep modulation by daytime CT-DBS in severe brain injuries and suggest a novel mechanistic framework for alpha-delta sleep generation across pathophysiologies.

Cheng et al. evaluated the effect of a sensory stimulation program (3 days per week for 4 weeks) using a ABAB design (for 16 weeks) in 29 patients. Higher CRS-R total scores were obtained during the treatment in the MCS group with increased arousal and oromotor function but not in the UWS group. Three patients also underwent fMRI and a modulation of brain activity related to treatment was found in specific brain regions.

In conclusion, this Research Topic offers some novelties in the field of severe brain injuries and DOC, including new techniques, methodology, diagnosis/prognosis improvements, and therapeutic options. We look forward to translate the neuroscientific evidence generated from these studies to the clinical context.

## Author Contributions

OG wrote the editorial. CS and SL revised it.

### Conflict of Interest Statement

The authors declare that the research was conducted in the absence of any commercial or financial relationships that could be construed as a potential conflict of interest.

